# Development and Validation of an Automated Video Tracking Model for Stabled Horses

**DOI:** 10.3390/ani10122258

**Published:** 2020-11-30

**Authors:** Nuray Kil, Katrin Ertelt, Ulrike Auer

**Affiliations:** Department of Anaesthesiology and Perioperative Intensive Care Medicine, Department for Companion Animals, Vetmeduni Vienna, 1210 Viena, Austria; Katrin.Ertelt@vetmeduni.ac.at (K.E.); Ulrike.Auer@vetmeduni.ac.at (U.A.)

**Keywords:** equine behaviour, image processing, automated video tracking, machine learning, pain assessment

## Abstract

**Simple Summary:**

Although there are some methods to detect pain in horses, because of bias and time-consumption, those methods are practically challenging. However, in recent years rapidly developed automated tracking methods have proven that computer-based behaviour monitoring is more reliable in many animal species. That is why in this study we aimed to investigate an automated video tracking model for horses in a clinical context. The findings will help to develop the automated detection of daily activity, to meet the ultimate objective of objectively assessing the pain and wellbeing of horses. An initial analysis of the obtained data offers the opportunity to construct an algorithm to track automatically behaviour patterns of horses.

**Abstract:**

Changes in behaviour are often caused by painful conditions. Therefore, the assessment of behaviour is important for the recognition of pain, but also for the assessment of quality of life. Automated detection of movement and the behaviour of a horse in the box stall should represent a significant advancement. In this study, videos of horses in an animal hospital were recorded using an action camera and a time-lapse mode. These videos were processed using the convolutional neural network Loopy for automated prediction of body parts. Development of the model was carried out in several steps, including annotation of the key points, training of the network to generate the model and checking the model for its accuracy. The key points nose, withers and tail are detected with a sensitivity of more than 80% and an error rate between 2 and 7%, depending on the key point. By means of a case study, the possibility of further analysis with the acquired data was investigated. The results will significantly improve the pain recognition of horses and will help to develop algorithms for the automated recognition of behaviour using machine learning.

## 1. Introduction

It is currently well established in veterinary medicine that pain triggers behavioural changes in animals, and their monitoring becomes relevant in the assessment of pain and in the evaluation of an animal’s welfare state. Detailed knowledge of both normal and pain-related behaviours in equines is imperative to properly evaluate pain. Although the presence of strangers or unfamiliar surroundings may mask pain-related changes, even subtle variations may become apparent if behaviour is thoroughly analysed [1].

In horses, pain is typically scored manually. Various pain assessment scales, such as the composite pain score [2,3] and the horse grimace scale [4], have been developed and proven useful in the assessment of postoperative pain. Horses have rich facial expressions based on 17 facial action units recently decoded [5]. Five of these units change in a typical manner during painful stimuli and interventions [4,6]. Composite pain scores include variables such as specific pain behaviour or posture that are scored using defined classes by means of simple descriptive scales [3,7]. A recently developed ethogram for pain scoring in ridden horses was introduced to determine musculoskeletal pain [8]. The fact that posture reflects the emotional state of a horse led to the attempt to use geometric morphometrics to assess back pain and poor welfare [9,10]. Yet, access by measuring activity patterns over a longer period, which changes in response to acute postoperative pain, seems to be more sensitive than composite pain scores [1].

All the methods have a limitation: they only observe the horse for a very short period. Executing repeated pain assessments increases the risk for noncompliance in clinics. Despite good validity for discrimination between not-in-pain and in-pain horses, inexperience in the common behaviour of a horse can lead to inconsistent results. This increases the risk of underestimation of pain or misinterpretation of behaviour in context of welfare and quality of life assessment.

Traditionally, behavioural studies and also activity pattern studies have been carried out manually or semi-manually with the limitation of subjective human observation [11,12,13]. Human observation to score behaviour imposes a number of limitations, such as the speed of scoring behaviour on video, the precision and recognizing a behavioural pattern that is rapid and variable [14]. Moreover, the manual analysis of such videos that were recorded over long periods (6 to 24 h) carries the disadvantage of being time-consuming and cumbersome. 

The introduction of computational ethology and biotelemetry overcomes these limitations [15,16]. Recent developments in computer vision, image processing and deep learning have improved research in behaviour sciences [17,18,19]. The developments in veterinary medicine in recent years have increasingly moved in the direction of replacing humans with their subjective and often non-reproducible assessments of behaviour—for example, regarding lameness in horses—with computer technology. Recently, studies on activity measurement and gait analysis using accelerometer data and convolutional neural networks have been published [20,21]. The positive results of these studies suggest that artificial intelligence should also be used for continuous observation of a horse’s behaviour. The use of video-based, automatic image analysis has the advantage of not only categorizing the behaviour automatically by means of an algorithm, but also of being able to review the video in case of ambiguities and deviations. These computer-aided analyses are not only interesting for questions in behavioural research but also for veterinary medicine for a more objective evaluation of pain and animal welfare.

The first video-tracking systems were introduced in the early 1990s. Due to their popularity for laboratory study, image analysis has been used extensively for video tracking of rodents under laboratory conditions [22]. Various automated methods—many prototypes—based on image subtraction, grayscale threshold, statistical models or colour tracking, have been used to study animal performance [22,23,24]. The advantage of image analysis is that it allows monitoring in a non-invasive way, without human presence or with limited human presence [25]. This is especially important for achieving high accuracy of pain assessments, as the animals feel unobserved and are less likely to mask pain behaviour.

The purpose of image analysis is to extract the information automatically to reduce manual workload, to increase objectivity and to quantify changes which are too slight for the human eye [26]. It allows one to study behaviours that occur briefly and are interspersed with long periods of inaction [27] or occur over many hours, such as diurnal variation.

Image analysis methods that have been developed so far for monitoring livestock, rodents and other animals are mostly based on “manual engineering of features,” also known as feature engineering (FE), followed by classification or regression modelling [27,28,29,30,31,32]. This differs from image analysis with deep learning techniques, developed recently for monitoring behaviour in different species [18,33,34,35,36]. Developments of deep learning techniques have introduced a new approach to image analysis in which feature engineering before classification or regression is not necessary, but rather, a “direct from video” approach is used. In this approach, the trained algorithm uses the raw video data to identify the animal and automatically detects the behaviour or action of an animal. 

The objective of this study was to evaluate how the video-based automatic tracking tool performs in the recognition of activity of stabled horses in a hospital setting. In addition, we give an example of how the deployment of the model could detect the behaviour of a horse in pain.

## 2. Materials and Methods

### 2.1. Horses

A total of 34 horses were used for this study. All horses were patients of the university’s equine teaching hospital and were housed in 3.5 meter-squared box stalls with free access to water, and roughage feed four times per day. Criteria for inclusion of horses in the study was different fur colour, fur badge, body size and shape and background, along with the incidence of light for the box. Informed owner consent was given prior to the start of the study. The university’s ethics committee and data protection committee approved the study.

### 2.2. Camera Setup and Video Recordings 

An action camera with a broad wide-angle (Gopro Hero 4, Gopro, Inc, San Mateo, CA, USA) mounted at a height of two meters in the right or left corner on the front side of the box stall was used. The camera was connected to a power bank for continuous power supply. Cameras were set up to see the whole area of the box stall. To reduce data space, recordings were taken in time-lapse mode (TLV) (two images per second) with a resolution of 2.7K. In total, 65 videos were used. In order to train and test the neuronal network, initially eight horses were taped over 24 h (in total 39 videos). Twenty-six horses were recorded only during daytime (in total 26 videos) and used for the validation process. 

### 2.3. Image Analysis 

For pose detection, we used the convolutional neuronal network Loopy (http://loopb.io, Loopbio GmbH, Vienna, Austria).

First, videos were uploaded to the server of Loopy and split into a training set of eight videos of eight different horses. The process of image analysis started with the labelling of markers on video files of 11.52 min corresponding to 21,342 images. In the selection of the video, the main focus was not on specific behaviour; instead, it was on the colour of the horse and the background of the box stall. Every 10th frame of the first 2500 frames (in total 249 frames per video) was chosen and prepared in the annotation section of Loopy. For each horse, the following anatomical landmarks were marked manually following the rules of good data in the documentation of the software. Three markers were annotated in each frame and the visibility of each marker classified as visible; 25, 50 or 75% occluded; or not visible. The annotated marker “wither” was defined as the highest point in the shoulder area where the neck merges into the back and the mane ends; “tail” was the area of the tail base; and “nose” meant the area including the mouth and nostrils.

The information in the dataset about the markers’ locations and whether they were visible or occluded, was entered into the deep-learning training tool for pose tracking to generate version 1 (V1)

Subsequently, with this version (V1) we ran a prediction on the remaining, not in the training set, used images. The results of the prediction are provided in Loopy as a video with the predicted markers, as a csv file for download and further analysis, and in a plot selection. The video generated by the software showed image by image the markers for nose, withers and tail. These markers were checked for correctness and mislabelling by means of visual inspection. Mislabelling was defined as a missing prediction of the visible markers over more than 30 consecutive images or repeated missing and repeated misplacement of a marker. Each sequence with misclassifications or missing predictions of visible markers over more than 30 consecutive images, or repeated missing and misplaced markers on wrong body parts or objects in the box, was identified. These sections in videos were manually re-annotated and incorporated in a new training process. 

### 2.4. Assessment of Robustness

The robustness of version (V2) was tested with the remaining unlabelled 31 videos of the 8 horses. After downloading the data with the pixel information of the markers, the numbers of images with predicted markers (PM) for nose, withers and tail (PMn, PMw, PMt) divided by the sum of all images (TI) per video were calculated. 

In this phase of the model creation, the misplacement of markers, defined as a difference of more than 200 pixels between the x and y pixels of each marker in two consecutive video frames, was checked mathematically. To detect these jumps the formula,

(1) $$\sqrt{{\left((X2-X1)\times (\mathrm{Frame}2-\mathrm{Frame}1)\right)}^{2}+{\left((Y2-Y1)\times (\mathrm{Frame}2-\mathrm{Frame}1)\right)}^{2}}$$

Was used and data were visually inspected and compared to the corresponding graphical representations. The number of wrongly predicted images, referred to as wrongly predicted (WP), was documented along with the reason for the mislabelling or non-labelled frames. 

Re-annotating of the mislabelled section was repeated and version 3 (V3) was generated with the additional annotated images. 

Model V3 was validated on 26 videos of different horses recorded during the daytime, which had not been annotated formerly. The mathematical analysis of mislabelled markers was repeated. A detailed performance analysis included the observation of the first and last 1000 images from the videos for counting each marker with the following classification: true-positive marker (TP = correctly predicted marker), true-negative marker (TN = correctly not predicted hidden marker), false-positive marker (FP = mislabelled or predicted but not visible) and false-negative marker (FN = visible but not predicted). With these figures, sensitivity was calculated utilizing the formula

TP/(TP + FN)(2)

To assess the error rate, FP/(TP + FP) was determined. The accuracy defined as the percentage of correctly classified markers (TP, TN) was calculated with the formula of (TP + TN)/(TP + FP + TN + FN). 

### 2.5. Statistical Data Analysis

For the comparison of the V2 and V3, a Student’s t-test was utilized for the total sum of PM, WP, the ratio of PMn, w, t/IE and PMn, w, t/WP for each video and marker. A one–way analysis of variance (ANOVA) was carried out to compare the quality of the prediction (sensitivity, error rate and accuracy) for all three markers. Statistical significance was accepted at *p* < 0.05. Statistical analysis was performed using NCSS 2020 (Statistical Software (2020)—NCSS, LLC, Kaysville, UT, USA, ncss.com/software/ncss.

### 2.6. Case Study to Demonstrate

Two videos of a horse recorded between 7 a.m. and 11 a.m. on day 2 post colic surgery and on postoperative day 8, respectively, were chosen to demonstrate the usability of the automated video tracking tool assessing the behaviour. A detailed analysis of the outcome parameter x and y pixel coordinates of the marker “nose” was done with the plot function in Loopy. 

A position scatter plot; a position heat map; a time spent in region calculation; and an x, y time series plot were generated and downloaded. The behavioural patterns observed with an ethogram, including behaviour classification for (1) standing observation, (2) sleeping or rest, (3) feeding and (4) moving, were scored image by image in the behavioural scoring tool in Loopy. 

Both the behavioural score and the x, y pixel value were downloaded as csv files. Both curves, behaviour score and timeline of x and y pixels, generated in Excel, were plotted together and represented in a graph. 

## 3. Results

**Figure 1:**
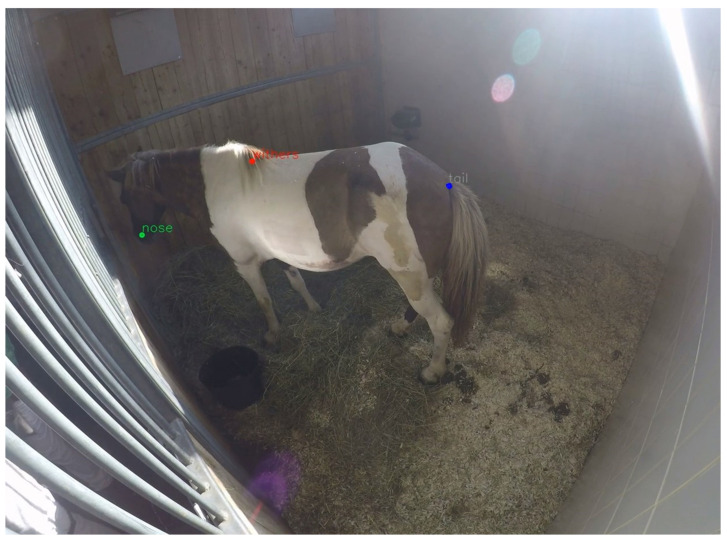
Screen shot of a video of the horse from the case example with the visible predicted marker nose (green), withers (red) and tail (blue).

The analysis of version 1, generated with 1990 images, revealed a continuous and accurate tracking of the three markers, at least in images similar to the annotated one, and a good resolution during movement of the horse (see Figure 1).

The visual video inspection of V1 resulted in reannotation and additional annotation of 1548 images in mislabelled sections to generate V2. The quality check of V2 revealed good overall performances for the markers “nose” and “withers” with mean PM/TI ratios of 0.7 and 0.4. The percentages of wrong markers were 1.1%, 3% and 2.8% for withers, tail and nose, respectively. In a number of 10 videos of four horses, video sequences with more than 2% WP for the marker withers could be detected. In 19 videos of five horses, the marker nose was predicted wrongly between 2 and 8% of the time. The percentage of WP for tail was similar to that of nose despite one horse with more than 20% WP in all five videos. 

The reasons for mislabelling were people’s interventions, lack of lighting conditions, horses’ angles to the cameras and blanket wearing. An additional annotation process increased the labelled images to 6244 (nose), 7276 (withers) and 5181 (tail). The resulting V3 performed with a significantly higher PM/TI ratio for all three markers and had significantly fewer wrongly predicted markers (see Table 1).

The mean values for sensitivity, accuracy and error rate of the 26 videos that were not included in the training process are given in Table 2, The detailed results per horse/video can be seen in Table S1.

### Case Example

**Figure 2:**
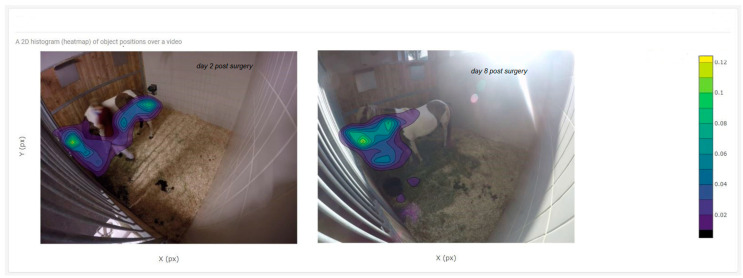
Heat map of the horse on day 2 and on day 8 post colic surgery. The horse spent 67% and 91% of the time in front of the box on days 2 and 8 respectively. Food was provided early in the morning and recording started at 06:30.

**Figure 3:**
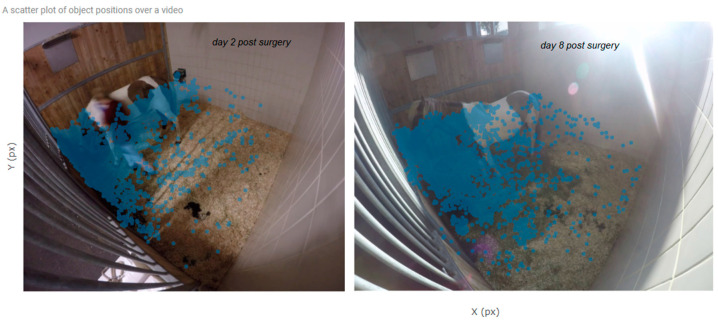
Position scatter plot of the horse on day 2 after colic surgery, and day 8. This plot gives additional information about the movement in the box over time in comparison to Figure 2.

The positions of the study horse during four hours in the mornings of days 2 and 8 postoperation are shown in Figure 2 as a heat map and in Figure 3 as a position scatter plot. The calculations of the preferred location in the box were different for the two days. On day two, the horse spent 33% of time looking to the sidewall compared to 9% on day eight.

**Figure 4:**
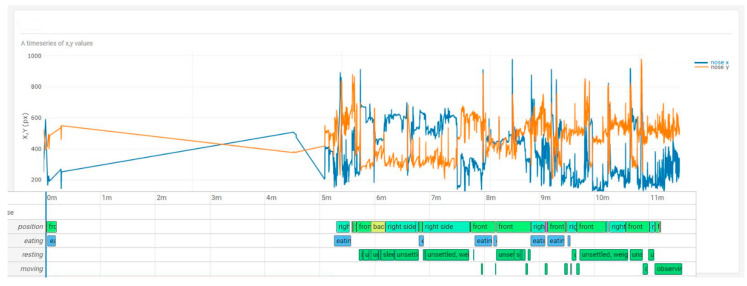
x, y time series plus behaviour score of the horse on day 2 post colic surgery. The oscillation mainly in the y pixel could be revealed as a behaviour during resting classified as unsettled standing with frequent weight shifting and extension of one forelimb while reducing weight on that limb.

**Figure 5:**
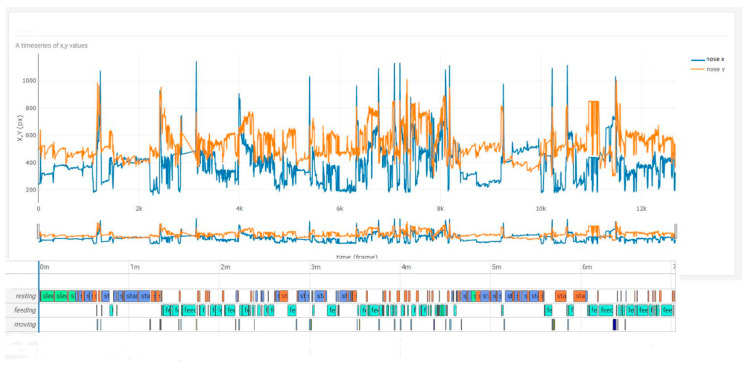
x, y time series plus behaviour score of the horse on day 8 post colic surgery. Weight shifting and extension of the forelimb was less frequent but still observable.

The comparison between the curve x for any pixels over four hours revealed a good accordance of the variance of x, y pixels with the corresponding behaviour score. (Figure 4 and Figure 5). A higher variance of mainly the y-coordinate during the resting periods could be also observed and seen in the video. This pattern could connected to an unsettled standing with frequent weight shifting and of the extension of one forelimb while reducing weight on that limb. This specific behaviour was seen continuously on day 2 during rest but was less noticeable on day 8. 

## 4. Discussion

This study presents a model for automated video tracking of horses to estimate behavioural changes in a clinical context. Annotating a small number of images was sufficient for the deep learning training of a predictor, which was able to mark three different body markers on time-lapse videos. The results revealed a high accuracy and sensitivity of more than 80% in pose estimation. An initial analysis of the obtained data offers the opportunity to construct an algorithm to automatically track the behavioural patterns of horses. 

Several open source video tracking software systems with special hardware requirements were recently reported [17,19,35]. The Web-based tool Loopy offers a complete set of image processing, tracking and behavioural analysis tools. The software includes a number of general-purpose algorithms for tracking the positions of subjects in videos. It offers custom training for a deep learning tracking solution for single and multiple animal-pose tracking. In the author’s opinion, the advantages are that Loopy is easy to use, does not need a special processor and computers requirements and always provides the latest technology in this area. As a user, no special knowledge in IT and coding languages is necessary. To generate a tracking model, an annotated ground-truth dataset for training that is generated with the annotation tool must be provided. The complexity of the scene, the visual similarity of individuals and a detection error tolerance determine how many training data are required to obtain a good result.

The decision to use the action cameras was based on their wide angle, flexible handling and easy installation. The quality of the video recordings and the time-lapse mode have proven to be suitable for generating the deep learning model and did not represent an obstacle for the prediction. Images can be recorded via simple camcorders [30], 3D cameras [36] or CCVT cameras [37]. Bonneau et al. (2020) used time-lapse cameras for object detection (goats in the field). The advantage of using action cameras in time-lapse mode is the low amount of memory required space [35].

In a first approach we decided to annotate only three markers on the horse. To have a better view of the behaviour of the horse, we decided to choose a camera angle of 60 degrees instead of a top down view. The markers for nose, withers and tail were almost always visible from this perspective. Only the tail marker was often not visible due to the preferred position of the horse with the head looking to the door, and therefore it was not predictable. The first version resulted in a nearly perfect prediction of all visible markers in images similar to the annotated one. In contrast to the position of the horse, the colour of the coat and the influx of the light had no negative effect on the prediction quality and accuracy in V1. The markers were also recognized during fast movement. An increase in the number of annotated images brought a clear improvement in terms of the position of the horse to the camera. With the second version we could concentrate on detecting the incorrectly labelled markers by means of mathematical analysis. This method, together with an optical control of the affected video section and the corresponding graphical representation, made it possible to identify the wrongly predicted marker. With this method, however, it is not possible to clearly distinguish between the false-positive predicted markers and the true-positive markers. The latter can show a greater distance between two images when the horse moves. We chose this method primarily to document an improvement between V2 and V3.

By further annotating images, we were able to achieve a significant reduction in the number of incorrect markers with version 3. The step-by-step procedure for improving the model is also described for other systems [38]. As was done here, it is also recommended that the videos be split into a training set and a test set—considered the gold standard in the development of deep learning models [18,19,34]. Following the recommendation of Graving et al. 2019 for machine learning models, we carried out the actual validation of V3 on videos that were not included in the annotation process [18].

Sensitivity and specificity are two statistical measures of the performance of classification test in the medical field but also for image processing [35,36,37,38,39]. Accuracy was used to evaluate the performance of automated activity tracking of goats using drones [40]. To the best of our knowledge, the present study is the first to deal with automated video tracking in horses. The values for accuracy and sensitivity are quite good and in line with the outcome of other deep learning tools [1,19]. Although the prediction rates of markers were low for some horses, additional annotation of the misclassified section and including in a new training model will improve the prediction for these horses. The main cause for misclassification was found to be the horses’ special features. For example, one of the horses had a brown coat colour but the nose area was partly brown and white; that is why the marker was classified as a false-negative. Automatic waterers, piles of manure and shoes belonging to people were often marked as a false-positives, especially in V2. However, further improvement of the model largely solved this mislabelling with V3. In general, however, it must be questioned whether incorrect predictions are not tolerable to a certain extent. The image resolution of our time-lapse video was two images per second. This means that 90 images of the TLV correspond to a real time of one minute. Mislabelling of a few images will have little influence on further analysis of behaviour and calculation of activity time budgets.

No doubt, if the percentage of incorrect markings is high in future predictions due to special coat colour, position of the horse or background colour, these sections should be annotated, and further deep learning training should follow to improve the model. This procedure is also recommended for other tools and in the documentation of Loopy software.

Based on the case study, we could get insights into the possibilities that arise with the data resulting from the automated video tracking. For example, the position of a horse in the box in relation to the door can be determined over a longer period of time; thus, validation of this parameter that occurs in many composite pain scales is possible with this method [7,41]. With the help of manual video analysis, Price et al. (2003) were able to determine that horses in the postoperative phase were standing with their heads in the back of the boxes more [1]. On day eight after the colic surgery, our horse stood with its head to the sidewall much less often than two days immediately after surgery. The apparent variance of the x, y pixels clearly can be assigned to different behavioural categories. Special behaviour such as frequent weight shifting during rest could be detected with the marker nose. Additional annotation of other markers, such as the hooves or ears, will improve the observation of behaviour. The realization of an algorithm for automated behavioural recognition is very promising. The automated calculation of time budgets for behaviour classes will be possible without the need for manually scoring the behaviour. In a further step an activity time budget can be related to a pain score to test the hypothesis of Price et al. (2003): whether this approach to pain recognition is more sensitive than pain scores [1].

The limitations in this study are primarily to be found in the video recording. For a continuous evaluation of the behaviour during the night, cameras with infrared technology are better suited than the action cameras chosen here. This limited the possibility of calculations within 24 hour time budgets.

## 5. Conclusions

The results demonstrate that the automated tracking of body parts using time-lapse videos is possible. The information generated with the deep learning module can be used to develop an algorithm for the automated classification of horse behaviour with high accuracy and resolution in a clinical setting. In the long term, this technology will not only improve the detection of acute and chronic pain in veterinary medicine, but also provide improved and new insights for behavioural research in horses.

## Figures and Tables

**Table 1:** Comparison of version 2 and version 3 in regard to total sums of predicted markers (PM) and wrongly predicted markers (WP), the ratio (PM/TI) of PM divided by total image (TI) and the percentage of wrongly predicted markers (%WP) in relation to number of predicted markers per video given as mean ± SD. Presented are data of the 39 training and test videos.

Version	Marker	TI	PM	WP	PM/TI	% WP
V2	Withers	704,608	471,544	7813	0.7 ± 0.2	1.8 ± 1.5
	Tail		288,499	4366	0.4 ± 0.2	3 ± 2.8
	Nose		511,817	12,908	0.7 ± 0.2	2.8 ± 2.3
V3	Withers	704,608	611,183	2561	0.9 ± 0.2	0.4 ± 0.3
	Tail		495,963	2985	0.7 ± 0.2	1.2 ± 2.2
	Nose		542,390	7541	0.8 ± 0.3	1.5 ± 1

**Table 2:** Mean ± SD of performance criteria for machine vison technics.

Marker	Sensitivity	Accuracy	Error Rate
Withers	0.88 ± 0.2	0.82 ± 0.3	0.02 ± 0.03
Tail	0.79 ± 0.3	0.82 ± 0.2	0.06 ± 0.20
Nose	0.94 ± 0.1	0.94 ± 0.1	0.07 ± 0.17

## Supplementary Materials

The following are available online at https://www.mdpi.com/2076-2615/10/12/2258/s1, Table S1: Detailed results of the counting first and last 1000 images per horse video. TN = true negative, TP = true positive, FN = false negative, FP = false positive.
