# Supplementary material for: Development and Validation of an Automated Video Tracking Model for Stabled Horses
Source: Animals (Basel). 2020 Nov 30;10(12):2258. doi: 10.3390/ani10122258 (PMC7760072; doi:10.3390/ani10122258)
Supplement: Supplementary file 1 [file animals-10-02258-s001.pdf]

Table S1: Detailed results of the counting first and last 1000 images per horse video. TN = true negative, TP = true positive, FN = false negative, FP = false positive.

Horse number	Marker	TN	TP	FN	FP	Sensitivity	Accuracy
1	Nose	144	777	74	5	0.91	0.92
2		0	978	21	1	0.98	0.98
3		63	903	10	24	0.99	0.97
4		81	673	169	77	0.80	0.75
5		65	910	19	6	0.98	0.98
6		1	989	10	0	0.99	0.99
7		36	929	33	2	0.97	0.97
8		0	1000	0	1	1.00	1.00
9		11	989	0	0	1.00	1.00
10		33	928	32	7	0.97	0.96
11		116	836	46	0	0.95	0.95
12		71	862	36	21	0.96	0.94
13		13	984	3	0	1.00	1.00
14		0	976	21	2	0.98	0.98
15		120	821	53	10	0.94	0.94
16		119	861	17	3	0.98	0.98
17		65	925	4	5	1.00	0.99
18		20	932	7	30	0.99	0.96
19		276	545	120	86	0.82	0.80
20		0	997	0	3	1.00	1.00
21		290	653	57	25	0.92	0.92
22		176	764	58	1	0.93	0.94
23		155	1561	273	11	0.85	0.86
24		263	968	714	55	0.58	0.62
25		134	1799	36	31	0.98	0.97
26		12	1987	0	1	1.00	1.00
1	Withers	0	2000	0	0	1.00	1.00
2		10	972	18	0	0.98	0.98
3		0	280	0	1720	1.00	0.14
4		0	1621	0	379	1.00	0.81
5		0	1117	732	151	0.60	0.56
6		0	2000	0	0	1.00	1.00
7		1	1807	133	59	0.93	0.90
8		0	1994	6	0	1.00	1.00
9		30	1922	48	0	0.98	0.98
10		11	306	669	14	0.31	0.32
11		0	1000	0	0	1.00	1.00
12		32	1104	639	225	0.63	0.57

13		8	1887	98	7	0.95	0.95
14		0	1957	38	5	0.98	0.98
15		0	1969	7	24	1.00	0.98
16		0	1998	2	0	1.00	1.00
17		0	1995	0	5	1.00	1.00
18		19	1764	77	140	0.96	0.89
19		3	337	630	30	0.35	0.34
20		70	1815	114	1	0.94	0.94
21		0	1680	312	8	0.84	0.84
22		4	1884	67	49	0.97	0.94
23		0	1338	225	437	0.86	0.67
24		14	1756	174	56	0.91	0.89
25		1	1292	674	33	0.66	0.65
26		0	1906	89	5	0.96	0.95
1	Tail	0	2000	0	0	1.00	1.00
2		0	0	1000	0	0.00	0.00
3		297	1682	9	12	0.99	0.99
4		410	1579	6	5	1.00	0.99
5		61	1389	547	3	0.72	0.73
6		0	1994	6	0	1.00	1.00
7		2	1011	985	2	0.51	0.51
8		11	1978	11	0	0.99	0.99
9		112	1714	174	0	0.91	0.91
10		661	20	166	153	0.11	0.68
11		434	260	306	0	0.46	0.69
12		840	584	543	33	0.52	0.71
13		252	1716	30	2	0.98	0.98
14		0	1919	11	70	0.99	0.96
15		0	1979	21	0	0.99	0.99
16		0	1997	3	0	1.00	1.00
17		0	1981	19	0	0.99	0.99
18		0	1980	17	3	0.99	0.99
19		329	546	85	40	0.87	0.88
20		0	1811	181	8	0.91	0.91
21		1161	817	0	22	1.00	0.99
22		226	1482	288	4	0.84	0.85
23		0	1435	412	153	0.78	0.72
24		7	997	507	489	0.66	0.50
25		79	867	1046	8	0.45	0.47
26		0	1997	3	0	1.00	1.00
